# Current Understanding of Immune Thrombocytopenia: A Review of Pathogenesis and Treatment Options

**DOI:** 10.3390/ijms25042163

**Published:** 2024-02-10

**Authors:** Alina Mititelu, Minodora-Cezarina Onisâi, Adrian Roșca, Ana Maria Vlădăreanu

**Affiliations:** 1 Department of Hematology, Carol Davila University of Medicine and Pharmacy, Emergency University Hospital of Bucharest, 050098 Bucharest, Romania; minodorel@yahoo.com (M.-C.O.); anamaria.vladareanu@umfcd.ro (A.M.V.); 2 Department of Physiology, Carol Davila University of Medicine and Pharmacy, 050471 Bucharest, Romania; adrian.rosca@umfcd.ro

**Keywords:** ITP, pathogenesis, rituximab, TPO-RA, splenectomy, SYK inhibitors, platelet desialylation

## Abstract

The management of immune thrombocytopenia (ITP) and the prediction of patient response to therapy still represent a significant and constant challenge in hematology. ITP is a heterogeneous disease with an unpredictable evolution. Although the pathogenesis of ITP is currently better known and its etiology has been extensively studied, up to 75% of adult patients with ITP may develop chronicity, which represents a significant burden on patients’ quality of life. A major risk of ITP is bleeding, but knowledge on the exact relationship between the degree of thrombocytopenia and bleeding symptoms, especially at a lower platelet count, is lacking. The actual management of ITP is based on immune suppression (corticosteroids and intravenous immunoglobulins), or the use of thrombopoietin receptor agonists (TPO-RAs), rituximab, or spleen tyrosine kinase (Syk) inhibitors. A better understanding of the underlying pathology has facilitated the development of a number of new targeted therapies (Bruton’s tyrosine kinase inhibitors, neonatal Fc receptors, strategies targeting B and plasma cells, strategies targeting T cells, complement inhibitors, and newer TPO-RAs for improving megakaryopoiesis), which seem to be highly effective and well tolerated and result in a significant improvement in patients’ quality of life. The disadvantage is that there is a lack of knowledge of the predictive factors of response to treatments, which would help in the development of an optimized treatment algorithm for selected patients.

## 1. Introduction

Immune thrombocytopenia (ITP) is an acquired autoimmune disorder characterized by a low platelet count (platelet count of less than 100,000/µL) due to an unbalanced interaction between effective and regulatory immune cells, resulting in an increased platelet clearance along with an impairment of thrombopoiesis [1,2]. It is relatively common and affects both adults and children, with an incidence in Europe ranging from 1.6 to 3.9 per 100,000/year [3,4] and a prevalence ranging from 9.5 to 23.6 per 100,000 adults. The incidence is higher among women than among men, but this is reversed in older patients [5]. In children, it occurs in about 5–10 per 100,000 children per year [4].

ITP is a condition that may be transient or chronic, and it is classified by The International Working Group in ITP as primary or secondary depending on the existence of an apparent precipitating factor or a predisposing condition [1]. In adults, 80% of newly diagnosed patients have primary ITP, which is characterized by isolated thrombocytopenia. Secondary ITP is triggered or associated with another disease, such as a chronic infection (Helicobacter pylori, human immunodeficiency virus—HIV, hepatitis C virus—HCV, or cytomegalovirus—CMV), an autoimmune disease (lupus erythematosus, antiphospholipid syndrome, rheumatoid arthritis, or an immunodeficiency state), a hematological condition (chronic lymphocytic leukemia, large granular lymphocytic leukemia, lymphoma, or autoimmune lymphoproliferative syndrome), or following therapy with drugs such as heparin and quinidine [5,6,7,8,9,10,11]. In children, it often occurs after a viral infection and is self-limiting in 80% of cases. In contrast, in adult patients, primary ITP develops into a chronic disorder in approximately 75% of cases [2].

Based on disease duration, ITP is classified as newly diagnosed (0–3 months), persistent (>3–12 months), and chronic (>12 months) [12].

The nature of ITP guides the choice of treatment. While thrombocytopenia in patients with secondary ITP may respond to treatment of the underlying disease, the situation is quite different in primary ITP [13]. According to the 2019 International Consensus Report on the investigation and management of ITP (ICR) and the American Society of Hematology (ASH) guidelines, patients should be treated based on their need rather than the disease stage, and patients’ quality of life became an important issue to be considered [12,14].

Corticosteroids remain the first choice for newly diagnosed ITP patients, but immunosuppression should be limited, a lesson learned even more profoundly during the COVID-19 pandemic. The use of newer agents, such as thrombopoietin receptor agonists (TPO-RAs), rituximab, and fostamatinib, as a subsequent treatment is supported by robust evidence and offers a viable alternative to splenectomy. Advances in deciphering the pathogenesis of ITP have facilitated the development of a number of new targeted therapies, such as those based on the inhibition of Syk inhibitors, Bruton’s tyrosine kinase (BTK), neonatal Fc receptors, or the complement pathway. Along with TPO-RAs, newer treatments will improve ITP care.

## 2. Pathogenesis

**Figure 1:**
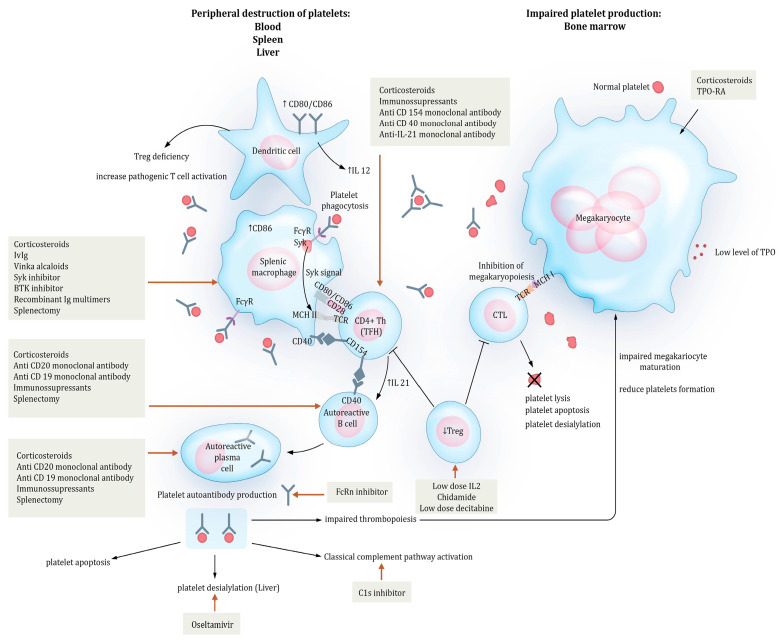
Pathogenesis of immune thrombocytopenia and targets for drug action. Immune thrombocytopenia results from peripheral destruction and impaired megakaryopoiesis. The key event is thought to be the production of antiplatelet antibodies that target platelet glycoproteins (GPs), thereby leading to platelet destruction. Platelets coated with antiplatelet antibodies are mainly destroyed in the spleen by splenic macrophages, and this is based on an Fcγ-dependent mechanism, which is controlled by spleen tyrosine kinase (Syk). Antigens from phagocytosed platelets are presented by the major histocompatibility complex class II (MCHII) to T-cell receptors (TCRs), which stimulates autoreactive T cells, i.e., follicular T cells (TFHs). TFHs interact with autoreactive B cells through the CD40/CD154 axis and IL21 production, promoting the differentiation of B cells into autoreactive plasma cells that will produce more antiplatelet antibodies. This autoimmune response is maintained by a deficiency in regulatory T cells (Treg). Opsonized platelets could also activate the classical complement pathway, and desialylated platelets are destroyed in the liver via an Fcγ-independent mechanism, thus promoting peripheral platelet destruction. CD8 cytotoxic T cells (CTLs) can mediate cytotoxicity against platelets, leading to platelet apoptosis, platelet lysis, or platelet desialylation. Impaired platelet production results from an autoimmune response targeting megakaryocytes (both antiplatelet antibodies and CTLs) and inadequate levels of thrombopoietin (TPO), which is the main growth factorof thrombopoiesis. The targets of action of current treatments and some molecules under investigation (not yet approved) are mentioned in this figure by red arrows. Abbreviations: BTK—Bruton’s tyrosine kinase; IL—interleukin; FcγR—IgG Fc receptor; FcRn—neonatal Fc receptor; IVIg—intravenous immunoglobulin; TPO-RA—thrombopoietin receptor agonist; C1s inhibitor—monoclonal antibody targeting C1s; CD—cluster of differentiation.

Immune thrombocytopenia is an autoimmune disease with a very complex pathogenesis. Primary ITP arises from several different mechanisms [15], such as the peripheral destruction of platelets opsonized by antiplatelet antibodies [16], impaired thrombopoiesis [17], and the T-cell-mediated destruction of platelets [18,19]; each pathogenic mechanism plays an independent role in generating thrombocytopenia (Figure 1).

Peripheral destruction of platelets occurs in the blood, spleen, and liver and, together with impaired bone marrow production, results in an autoimmune response against megakaryocytes and peripheral thrombocytopenia [20].

Persistence of this autoimmune response is favored by a loss of tolerance, which is supported by a deficiency of regulatory T cells (Tregs) in the spleen, blood, and bone marrow, with defects in the B and T cells, leading to both pathological autoantibody formation and abnormal T-cell responses [21].

The pathogenesis of secondary ITP may share similar mechanisms with primary ITP. However, unique mechanisms have been identified in some types of secondary ITP. For example, antigen mimicry, in which antibodies directed against a foreign protein cross-react with specific epitopes on platelet glycoproteins (GPs), has been observed in thrombocytopenia associated with hepatitis C, Helicobacter pylori, and HIV infections [7,22].

### 2.1. Mechanisms of Peripheral Destruction

Antibody-mediated cytotoxicity relies on various mechanisms.

In blood, IgG antiplatelet antibodies target platelet surface glycoproteins (GPs), mainly GPIIb/IIIa (fibrinogen receptor) and GPIb/V/IX (von Willebrand receptor), and less commonly GPIa/IIa (collagen receptor), GP IV, and GPVI [23]. Antibody-coated platelets are removed from circulation via phagocytosis in the splenic reticuloendothelial system, and by splenic macrophages through an FCγ receptor (FCγR)-dependent mechanism [23].

Antiplatelet antibodies are produced by B-cell clones harboring a hypermutation in both heavy- and light-chain gene usage [24], which are stimulated by the interaction with T follicular helper (TFH) cells. They are found in different places starting from the spleen, which has an expanded and most likely dysregulated germinal center, blood, and bone marrow. Long-lived plasma cells are also produced in the process of antibody secretion [23].

In ITP, macrophages are both effector cells and antigen-presenting cells (APCs). Splenic macrophages are the major antigen-presenting cells that stimulate autoreactive CD4 T-helper cells (Th) in ITP [16]. These autoreactive Th cells contain TFH cells that interact with autoreactive B cells and induce their proliferation and differentiation into plasma cells, with the production of antiplatelet antibodies, through a mechanism dependent on IL-21 secretion and CD40/CD154 interactions [18]. B-cell activating factor (BAFF) is a cytokine produced by monocytes (and polymorphonuclear cells) that participates in the stimulation and survival of B cells and plasma cells [25]. Regulatory B cells (Bregs) are involved in maintaining peripheral tolerance by secreting IL-10, through which they recruit Tregs and reduce the function of autoreactive Th cells [26]. The increased number of autoreactive plasma cells, together with high levels of BAFF, has been strongly associated with ITP [16]. Peripheral tolerance in B-cell compartments promoted by Bregs is impaired in ITP [16].

As effector cells, macrophages contribute to platelet destruction. The FCγR family is composed of activating receptors (FcγRI, FCγRIIa/c, and FCγRIII) and inhibitory receptors (FCγRIIb). Most cells express both types and can trigger their activation depending on their relative expression. During ITP, monocytes have an increased expression of FcγRI and FCγRIIa/FCγRIIb ratio, which is associated with an increase in their phagocytic capability [23].

Dendritic cells (DCs) participate in triggering the immune response as monocyte-derived dendritic cells. They are able to phagocytose apoptotic platelets and stimulate T cells. They have tolerogenic capacities and are involved in the conversion of naïve T cells into regulatory T cells. They are also involved in modulating T-cell polarization toward Th2 commitment [27]. In ITP, they appear to express larger co-stimulatory molecules CD86 and CD80, and they produce increased amounts of IL-12. These modifications are correlated with Treg deficiency and an increase in pathogenic T-cell activation [23].

The involvement of complement in platelet clearance is observed in 50–60% of cases, and it is correlated with the type of antiplatelet antibody; anti-GPIIb/IIIa is more susceptible to the activation of the complement pathways than anti-GPIb/IX (73–100% versus 40–65%) [28].

Complement activation leads to C3b deposition on the platelet surface, which promotes the phagocytosis of opsonized platelets by macrophages in the spleen, a process mediated by the ligation of antiplatelet antibodies to FCγR, and C3b to CR1 [19].

An FCγR-independent pathway of platelet destruction is platelet desialylation. Studies have shown that GPIb/IX antibodies trigger platelet desialylation by recruiting neuramidase-1 to the plasma membrane [29]. Desialylated platelets are recognized by the Ashwell–Morell receptor expressed on hepatocytes, leading to their removal from circulation and to the production of TPO, a major growth factor of megakaryocytes [29].

### 2.2. T-Cell Involvement

In ITP, effector immunity consists of autoantibodies and T cells. Dysregulation of T cells plays a crucial role in the pathogenesis of ITP.

Due to a breakdown of self-tolerance, the APC process is triggered and presents platelet autoantigens to autoreactive T cells, which initiates a cascade of events including the stimulation of autoantibody production and cytotoxic T-cell (CTL) activation and proliferation, along with an abnormal number and function of regulatory T cells (Tregs), the production of abnormal Th cells, and abnormal T-cell anergy [30].

Tregs ensure immune tolerance by regulating B- and T-cell-mediated autoimmunity and induce a tolerogenic phenotype through interaction with DCs. A loss of peripheral tolerance is due to the suppression of the activity of regulatory T cells, which is determined by a decreased generation of and/or an alteration in the function of these cells [19,30,31]. The degree of Treg abnormalities is associated with disease severity [22]

CD4 T-helper cells play a crucial role in the differentiation of B cells into autoantibody-secreting plasma cells. Multiple Th populations (Th1/Th17/Th22/Treg) contribute to the pathogenesis of ITP, and proinflammatory Th17 cells are important players in the development of autoimmunity [32]. TFHs are important players in the pathogenesis of ITP. They express the CD154 surface receptor and are the main producers of IL-21, supporting the differentiation of autoreactive B cells and the production of antiplatelet antibodies [33]. A Th1/Th2 imbalance is inversely corelated with disease severity [34].

CD8 cytotoxic T cells can mediate cytotoxicity against platelets; however, due to the necessity of a close cell contact, this phenomenon probably occurs in the spleen [19]. Moreover, there is a recruitment of CTLs into the bone marrow where they participate in the immune response against megakaryocytes [19].

The presence of CTLs is consistent with an increased cytokine imbalance toward IL2 and interferon (IFN), indicating a shift toward Th1 cells. In ITP, type1/type2 ratios are unbalanced, with high Th1/Th2 ratios and high Tc1/Tc2 (cytotoxic CD8 + cells) ratios [22,30,35]. Moreover, CTLs seem to have a direct role in platelet and megakaryocyte lysis in bone marrow [35].

The involvement of natural killer (NK) cells in ITP pathogenesis has been scarcely described, and it seems less likely that these cells are involved [19,36,37].

### 2.3. Impaired Thrombopoiesis

Impaired thrombopoiesis with an insufficient production of platelets is the result of an abnormal immune response against megakaryocytes (MKs), which is associated with a low TPO concentration [38]. In ITP, since MKs express the same GP as platelets, they are clearly targeted by antiplatelet antibodies that bind to GPIb and GPIIb/IIIa, as well as by CTLs, which induces morphological and physiological changes or even apoptosis [16,19,23,39].

In addition, MKs have been observed to have an intrinsic impairment in platelet production and a decreased ability to generate proplatelets [40].

Platelet production is correlated with the serum levels of TPO, a major megakaryocyte growth factor. TPO is produced by the liver during the physiological process of platelet senescence. Senescent platelets undergo a desialylation process and are recognized by the Ashwell–Morell receptor (AMR), leading to their phagocytosis with TPO production through the JAK/STAT3 signaling pathway in a negative loop [41]. In thrombocytopenia, the amount of free TPO that stimulates MKs is high. During ITP, the production of platelets is close to normal, with a very short lifespan, which translates into a lower TPO concentration. This insufficiency is reversed with the use of TPO-RAs [38].

Moreover, immature MKs and mesenchymal stem cells (MSCs) are affected and appear to be apoptotic, which is associated with a decreased ability to stimulate Treg proliferation and IL10 production [23].

## 3. The Role of Platelets in ITP

In addition to being the main target of effector immunity, it has been reported in recent years that platelets exhibit some non-hemostatic immunological functions, being active cells involved in ITP pathogenesis. They are the scavengers of the immune system and immune effectors, which are capable of modulating both innate and adaptive immunity [42].

Platelets can initiate the antimicrobial host defense by acting as immune cells in the presence of pathogens or inflammation through multiple immune receptors (immunoglobulin or complement receptors) and Toll-like receptors (TLRs) [43].

For platelets to carry out their immunological function, they need to be activated.

So far, although a significant proportion of autoantibodies that target platelet glycoproteins are capable of triggering platelet activation, by interacting with glycoproteins such as GPIIb/IIIa or GPIb/IX/V, there are conflicting results about platelet activation in ITP [42]. As immune cells, platelets are active participants of the immune responses in ITP by expressing low levels of CXCL5, CCL5, EGF, and CD40L [15,22], whichappear to be strongly associated with the degree of thrombocytopenia [44].

## 4. Current Management of ITP

### 4.1. Whom to Treat

In adults, the goal is to maintain a hemostatic platelet count while minimizing the toxicity of therapy [12,45]. Currently, there are no clinical trials to demonstrate the superiority of any treatment option and there is significant variability regarding the therapeutic approach. Current guidelines recommend the observation of asymptomatic patients with mild or moderate thrombocytopenia and individualized treatment related to the disease phase for patients at a higher bleeding risk and with a low platelet count (below 30,000/µL) [12,45]. Age, degree of bleeding, comorbidities predisposing patients to bleeding, complications of specific therapies, patient expectations, lifestyle and quality of life, tolerance of side effects, and a patient’s need for non-ITP medication that may create a bleeding risk are important factors that contribute to the treatment decision [12,45].

### 4.2. Bleeding Risk

The clinical manifestation of bleeding in ITP patients ranges from minor to severe and may be localized on the teguments, oral cavity, gastrointestinal tract, pulmonary tract, urinary tract, or genital tract, or manifested as intracranial hemorrhage.

Based on findings from ITP registries and administrative databases, as well as clinical studies, the frequency of severe bleeding, manifested as intracranial hemorrhage (ICH), is ∼0.5% in children and 1.5% in adults [46,47]. Only a small proportion of patients with ITP develop severe bleeding.

Bleeding risk is not directly correlated with platelet count, but a target level of 20–30 × 10^9^/L is suggested [12,45].

Predictors of severe bleeding, as evaluated in clinical trials, include the presence of severe thrombocytopenia (platelet count <10,000–20,000/µL) [48], previous minor bleeding [49], older age (older than 60 years) [50], concomitant medication use that predisposes patients to bleeding, and male sex [47]. Response to TPO-RA treatment is associated with an improvement in bleeding outcome [51,52].

There are several tools to assess bleeding that are specific to patients with ITP, of which the Buchanan tool for children and the Page score for adults have been most widely used [53].

The Page score, also called the ITP Bleeding Scale (IBLS), evaluates the severity of bleeding and comprises 11 grades scored on a scale from 0 (no bleeding) to 2 (marked bleeding). Bleeding at nine anatomical sites (cutaneous, oral, epistaxis, gastrointestinal, urinary, gynecological, pulmonary, intracranial, and subconjunctival) is assessed based on patient history, while bleeding at two of these sites (cutaneous and oral) is assessed by means of physical examination [53].

Assessing and understanding the risk of bleeding, as well as the underlying determinants of bleeding, can help detect patients who may require therapy. Physicians should also evaluate the necessity of treatment, toxicity, and dose limitation for chronic ITP; combination therapy for severe bleeding associated with thrombocytopenia; and early aggressive therapy that may result in a durable platelet count response [47,54].

Therefore, the treatment decision should not be dictated by the platelet count alone. It should consider other important factors such as the individual bleeding phenotype of each patient, lifestyle, comorbidities, medication affecting hemostasis, the need for concomitant antithrombotic therapy, and the need for an invasive procedure or surgery. Patient preferences, although not very well defined in the latest guidelines, also constitute a very important factor to consider. Activity level, pregnancy planning, disease anxiety (low platelet count, bleeding tolerance, and cosmetic appearance of bruises/petechia), and adherence to treatment (long-/short-term treatment vs. splenectomy, injection vs. tablets, and daily vs. weekly administration) are some of the patient preferences defined in these guidelines that should guide the treatment selection [55].

### 4.3. How to Treat

Treatment indications have changed considerably recently. According to the ICR and ASH guidelines for ITP published in 2019, treatment options are now divided into initial, subsequent, and surgical treatments [12,45].

Treatment options are different according to the phase of the disease (newly diagnosed, persistent, or chronic), but, overall, the clinical goal should be to resolve bleeding events or to prevent severe bleeding by ensuring adequate hemostasis [1].

The degree of thrombocytopenia in ITP is the result of suboptimal platelet production, together with increased platelet destruction induced by immune dysregulation.

**Figure 2:**
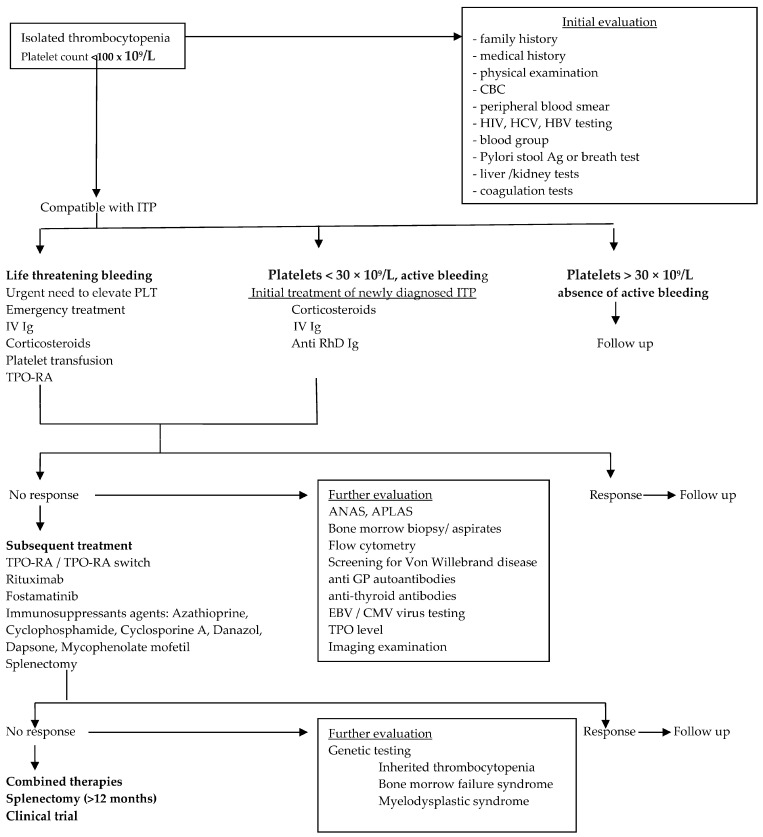
Therapeutic approach for patients with ITP. The diagnosis of ITP is first evaluated by excluding non-autoimmune causes of thrombocytopenia and secondary ITP. After diagnosis, the need for treatment is assessed. Follow-up is recommended for patients with platelet counts above 20,000 to 30,000/µL and no bleeding. For patients requiring therapy, the first option is administration of corticosteroids, IVIg, anti-D, and platelet transfusion (in emergency situations and for selected patients, TPO-RA). Rare cases of secondary thrombocytopenia are ruled out at first relapse, and one of the options for subsequent treatment is chosen. A lack of response to treatment calls for additional evaluation with genetic testing. In terms of treatment, patients and physicians can choose between combined therapies, innovative therapies being evaluated in ongoing clinical trials, or splenectomy if it has not already been performed. Abbreviations: ITP—immune thrombocytopenia; CBC—complete blood count; HIV—human immunodeficiency virus; HCV—hepatitis C virus; HBV—hepatitis B virus; plt—platelets; IVIgs—intravenous immunoglobulins; TPO—thrombopoietin; TPO-RA—thrombopoietin agonist receptor; ANAs—antinuclear antibodies; APLAs—antiphospholipid antibodies; GP—glycoprotein; EBV—Ebstein Barr virus; CMV—cytomegalovirus.

Current treatment of ITP attempts to either increase the level of TPO (TPO-RA) or decrease the immune response (corticotherapy, CD20-targeted monoclonal antibodies, immunoglobulins, immunosuppression, and splenectomy), or both [56,57] (Figure 2).

Steroid treatment remains the cornerstone of first-line therapy, but this should be used for a limited time. Two commonly used regimens are high-dose dexamethasone and oral prednisone/prednisolone (Table 1). Corticosteroids have multiple beneficial hemostatic effects. They can decrease platelet clearance and increase platelet production [58]. They also have a direct effect on blood vessels by means of which they may reduce bleeding, independently of platelet count [59]. Moreover, they may interact with immune cells to exert an inhibitory effect on macrophages, B and T cells, and dendritic cells [57]. Despite the fact that approximately 75% of patients initially respond to corticosteroids, the majority relapse during discontinuation [60]. There are limited data available, gathered through small studies that evaluated which steroids could provide a better response. Overall, it appears there are no differences regarding platelet count response at 6 months between prednisone and dexamethasone, although dexamethasone leads to faster responses without additional toxicity [49,61,62,63].

Intravenous immunoglobulins (IVIgs) may be added to the treatment of patients with a contraindication to steroids or those whose platelet counts decrease with steroid tapering [12,45]. These agents result in a response rate similar to corticosteroid therapy, but with a low duration of response (2–4 weeks) [39]. They are preferred as bridging agents and are used to maintain a stable platelet count until a more appropriate therapy can be initiated [12]. Although the full mechanisms are not yet completely understood, it appears that the Fc portion of IVIgs facilitates the clearance of autoantibodies by saturating the neonatal receptor (FcRn) and inhibiting the IgG-activating receptors (CD16/FCγRIII, CD32A/FCγR IIA, and CD64/FCγRI) [19]. They also appear to inhibit cytotoxic T-cell activation, neutralize complement activity, and inhibit megakaryocyte apoptosis [64,65]. Although IVIg as a standard ITP therapy is well documented, several studies have focused on the relationship between IVIg and the type of antiplatelet antibodies. A reduced IVIg response has been associated with anti-GPIb-IX antibodies, but this fact needs to be confirmed by further studies [66]. Aseptic meningitis, nephrotoxicity, thrombosis, fluid overload, and severe hemolytic anemia are the most commonly reported adverse reactions [12].

Anti-D immunoglobulin (only for Rh-positive patients with an intact spleen) is a useful agent, especially for children or patients with HIV-associated ITP. It binds to the Rh(D) antigen, leading to the clearance of antibody-coated red cells and, thus, a decrease in the clearance of opsonized platelets by the reticuloendothelial system [67]. Attention should be paid to the risk of intravascular hemolysis leading to death, anemia, multisystem organ failure, renal failure, and disseminated intravascular coagulation [68].

Rituximab is an anti-CD20 monoclonal antibody that triggers the apoptosis of CD20 lymphocytes via antibody-dependent cell-mediated cytotoxicity and complement-mediated lysis. It seems to trigger an initial response in approximately 60% of cases, with the response rate maintained at 40–60% one year later and at around 20–30% five years later [69]. Female sex, a shorter duration of disease, and age under 40 are associated with sustained responses [70]. It is worth mentioning that reactive plasma cells do not express CD20, and the persistence of long-lived plasma cells in the spleen or bone marrow has been associated with a lack of response to rituximab [71].

TPO-RAs (eltrombopag, romiplostim, and avatrombopag) have been shown to be effective in approximately 70–80% of patients, with responses maintained over time in around 50–60% of patients [72]. They act by binding to the TPO receptor on megakaryocytes, causing conformational change and activation of the JAK/STAT5 pathway with a consequent increase in platelet production [73].

Romiplostim is a peptide with four TPO receptor-binding sites linked to an IgG1-Fc component and mostly stimulates mature precursors. Eltrombopag is a small molecule that binds to a transmembrane site and acts earlier in the pathway by stimulating MK precursor cells and MK differentiation [73,74,75]. In addition to increasing platelet production, some immunomodulatory effects have been described [73]. This TPO-RA can increase regulatory T- and B-cell effects, mediated by the transforming growth factor beta (TGF-B), which is a major cytokine involved in Treg-cell development and is found in high levels in MKs and platelets [76].

These differences may explain the variation in response in patients with ITP [77,78,79].

Avatrombopag is a second-generation TPO-RA recently approved for patients with chronic ITP who have had an unsatisfactory response to other treatments [80]. It acts by binding to the transmembrane region of the TPO receptor and activates signal transduction pathways, leading to increased platelet production [80]. It has a high durable response rate (93% vs. 7% in the placebo group) and may also have a corticosteroid-sparing effect [80].

Thrombopoietic agents are well tolerated, with thrombotic risk remaining the major concern. Another aspect is that prolonged exposure can lead to an increase in reticulin deposits in the bone marrow [81]. Other reported side effects are headache, arthralgia, myalgia, dizziness, and hepatotoxicity [82].

Splenectomy has been the gold standard for many decades, mainly because of the high rates of durable remission [83]. Current IWG and ASH ITP guideline recommendations are to delay splenectomy for at least one year after initial disease onset and to offer it to patients who relapse or do not show a response to steroids; also, if possible, all medical treatments should be administered before surgery is considered [12,45]. With an initial response rate of up to 90%, and a median response rate of 78% at 10 years and 68% at 20 years, splenectomy is the only option associated with long-term treatment-free remissions [84]. Some studies attempted to correlate patients’ response to splenectomy with predictive factors such as age, sex, duration of disease, interval from diagnosis to splenectomy, and previous response to corticosteroids or IVIgs [85]. Of all the predictor variables that were tested before splenectomy, younger age was most often found to be associated with a good response [86]. The effectiveness of splenectomy is related to the removal of the platelet destruction site, where the autoimmune process is maintained. Some studies have evaluated the role of platelet scintigraphy as a promising predictive factor of splenectomy Although useful in identifying the site of platelet clearance, this method is not widely used [85,86]. Failure to respond to splenectomy may be explained by persistent platelet destruction in the liver and/or bone marrow, or the persistence of autoreactive plasma cells [83,87].

## 5. New Therapeutic Targets in ITP: From Pathophysiology to Treatment

Relapsed/refractory patients have prompted the need to identify new molecules that target different pathways involved in the pathogenesis of ITP. Phagocytosis inhibition targeting FCγR signaling (spleen tyrosine inhibitors and Bruton’s tyrosine kinase), neonatal Fc receptor inhibition (Rozanolixizumab, Nipocalimab, and Efgartigimob), inhibition of the classical complement pathway (Sutimlimab), and inhibition of platelet desialylation (Neuraminidase-1 inhibitor) are novel strategies to limit peripheral platelet destruction. New anti-CD20 monoclonal antibodies (veltuzumab and obinutuzumab) to limit autoreactive B cells, proteasome inhibitors, and therapies targeting BAFF (belimumab) to remove long-lived plasma cells in a BAFF-rich microenvironment appear to offer promising results. Anti-CD40 monoclonal antibodies target TFHs involved in ITP pathogenesis via the CD40L/CD40 axis and IL-21 production. The restoration of Treg function with low doses of Il-2 or histone deacetylase (clidamycine) has also been evaluated [19,57].

### 5.1. Inhibition of Phagocytosis by Targeting FCγR Transduction Signal

Recognition of opsonized platelets by splenic macrophages occurs via their FCγR receptors (FCγR I and FCγR III) [88]. After the cross-linking of immune complexes to FCγR, phosphorylation by Lyn (a Src family kinase) of the immunoreceptor Tyrosine-based Activation Motif (ITAM) domains contained in the cytoplasmic portion of FCγR occurs [88]. This step leads to the recruitment of Syk, an enzyme involved in the phosphorylation cascade that results in the activation of phagocytosis and cytokine production. Among the various recruited molecules, Bruton’s tyrosine kinase, which activates Rac and Rho, thereby leading to the reorganization of the cytoskeleton, is also required for phagocytosis. Syk is widely expressed in hematopoietic cells, immune cells (B cells, T cells, and macrophages), and platelets. By being expressed in B cells, Syk plays a role in antibody formation and represents an attractive target for ITP therapy [89].

Therefore, platelet phagocytosis by splenic macrophages could be targeted by spleen tyrosine kinase (Syk) or Bruton tyrosine kinase inhibitors.

Fostamatinib is a Syk inhibitor recently approved for the treatment of chronic ITP in patients that have failed at least one previous therapy, showing a response rate of around 43% in previously heavily treated patients, and a maintained response of around 18% [19]. The most common adverse reactions are diarrhea, hypertension, and nausea [90].

Bruton’s tyrosine kinase is another molecule involved in FCγR transduction signaling. As it is expressed in platelets, the inhibition of BTK also affects platelet aggregation. Rilzabrutinib is a modified BTK without an effect on aggregation that targets the underlying disease mechanisms of platelet destruction. It showed promising results in a phase I/II study, with a 44% response rate in 32 intensively pretreated chronic ITP patients, regardless of splenectomy or prior therapy [91]. A total of 71% of these patients achieved a significant platelet response (50,000/µL) that was maintained over time [19]. Rilzabrutinib is well tolerated, with transient side effects affecting mainly the gastrointestinal tract [91]. Both Syk and BTK contribute to B-cell receptor signaling and could affect antibody production [92].

Neonatal Fc receptor (FcRn) could accelerate the clearance of antiplatelet antibodies [93]. FcRn is a member of the MHC class I family molecules. It is a homeostatic receptor that is expressed by endothelial, epithelial, and hematopoietic cells and plays an important role in extending the lifespan of IgG and albumin for up to 21 days. FcRn is able to bind to IgG and albumin in acidified endosomes and allows their release into the circulation, while unbound proteins are degraded in lysosomes [57,93].

Monoclonal antibodies—Rozanolixizumab, Nipocalimab, and Efgartigimob, which contains a modified Fc portion of IgG1—are FcRn inhibitors with good tolerance and a response rate of approximately 38% (for Efgartigimob) and 50% (for Rozanolixizumab) in ITP-pretreated patients. They produce a rapid reduction in IgG concentration, without affecting the concentration of IgM, IgA, and albumin [57].

Recombinant Ig multimers are under evaluation, with promising results in animal models. Even though IVIgs are highly efficient in the treatment of ITP, their blood-derived origin could lead to immune side effects, which could be avoided by using the abovementioned new molecules.

#### 5.1.1. Inhibition of Complement

The involvement of complement in the pathogenesis of ITP through the activation of the classical complement pathway is a well-known fact. After opsonizing platelets with antibodies, the complement pathway becomes activated and leads to the deposition of C3b on the platelet surface, which facilitates the splenic phagocytosis of opsonized platelets by macrophages expressing the complement receptor (CR1) [18]. Complement activation also correlates with disease activity as it is higher in patients with an active disease [94]. However, it has only recently begun to gain attraction in the field of therapeutic research.

Sutimlimab has opened up the possibility of complement inhibition in ITP patients. In a study involving 12 patients with ITP, Sutimlimab acted by decreasing C3b deposition and preventing the formation of the membrane attack complex, showing a response rate of 42% and a complete response of 32% [95]. All patients achieved a durable complete response, with no significant side effects.

The downside is that complement activation is not involved in all patients with ITP, and there is an urgent need to identify biomarkers and determine which patients may benefit from treatment.

#### 5.1.2. Inhibition of Platelet Desialylation

Oseltamivir is a neuraminidase-1 inhibitor that is occasionally used in refractory ITP. Discovered accidentally, it showed its potential by improving the response to immunosuppressants or TPO-Ras in 10 out of 16 ITP patients with anti-GPIb/IX antibodies [96].

### 5.2. B-Cell- and Plasma Cell-Targeting Therapies

Targeting B cells and plasma cells could improve the reduction in pathogenic autoantibodies in patients with ITP.

#### 5.2.1. Anti-CD20-Targeting Therapies

In addition to rituximab, other anti-CD20 monoclonal antibodies have been developed, including veltuzumab and obinutuzumab. Despite their good tolerability and efficacy in depleting B cells in hematological B-cell malignancies, they are not routinely used in patients with ITP.

#### 5.2.2. Combination Therapies

Due to the persistence of long-lived plasma cells in a BAFF-rich microenvironment, the combination of rituximab with belimumab (a monoclonal therapy targeting BAFF) shows a promising response, with an 80% response rate in a study with 15 ITP patients [19].

#### 5.2.3. Plasma Cell-Targeting Therapies

The presence and persistence of long-lived plasma cells has increased interest in plasma cell-targeting therapies. Proteasome inhibitors (Bortezomib) may be useful in depleting autoreactive plasma cells. Similarly, monoclonal antibodies directed against clusters of differentiation expressed by plasma cells, such as CD38 (daratumumab and mezagitamab) or CD19 (inebilizumab), show efficacy in depleting clonal plasma cells and clonal B cells [71].

Mezagitamab acts by binding to CD38, thereby inhibiting enzymatic activity inducing apoptosis, cytolyzing cells via antibody-dependent cellular cytotoxicity and complement-dependent cytotoxicity, and reducing NK cells and sub-populations of B and T cells [72].

A major issue related to plasma cell depletion is the risk of hypogammaglobulinemia, which could aggravate serious infectious complications [57].

### 5.3. T Cell-Targeting Therapies

#### 5.3.1. CD40/CD154 Blockage

The CD40-CD154 (CD40L) axis acts as a costimulatory signal at different levels that promotes cell survival, cell differentiation, and cytokine production [97]. A tight regulation of the CD40/CD154 axis is necessary to maintain appropriate immune responses. Both CD40 and CD154 are expressed in a wide variety of immune cells (APCs, B cells, and T cells). APCs express CD40 through which they can stimulate autoreactive T cells, which express CD154. TFHs express CD154, thus participating in the activation of autoreactive B cells and the production of antiplatelet antibodies [97]. Platelets also express CD154 and they are supposed to directly activate autoreactive B cells and the production of antiplatelet antibodies [98].

The inhibition of the CD40/CD154 axis has been evaluated in refractory patients with ITP. Ruplizumab and toralizumab are anti-CD154 antibodies that show a good response rate, but also an increased rate of thrombosis, in animal models [19].

Letolizumab is an FC-modified anti-CD154 antibody that lacks the ability to bind to FCγR IIA expressed on platelets, but it binds to FcRn, thereby prolonging its half-life. It also appears to be able to increase Tregs and decrease memory T cells [57].

#### 5.3.2. IL-21 Inhibition

Given the mechanism of action of TFHs during B-cell stimulation, IL-21 blockade is another relevant field of investigation. IL21 is required, together with CD40-CD154 interaction, to activate clonal B cells. IL-21/Il-21R blockade shows reduced B- and T-cell activation with an interruption of disease progression [99].

#### 5.3.3. Treg Restoration

To restore tolerance by promoting Treg expansion or improving their function, some mechanisms such as the modulation of IL2 signaling or epigenetic modulation have been evaluated.

Modulation of IL-2 signaling is under investigation in several studies that include patients with ITP [57].

Chidamide is a histone deacetylase capable of enhancing the immunosuppressive function of Tregs and converting T cells into Tregs, leading to platelet correction [100].

Decitabine is a demethylating agent that showed a good response (51% response, 27% with complete response) in 51 patients with ITP who had undergone at least three prior lines of therapy [101]. Its mechanism of action appears to involve an enhancement of Treg function, a decrease in effector T cells, and the promotion of megakaryopoiesis [102].

### 5.4. Enhancement of Thrombopoiesis

The newer generation TPO-RAs, such as avatrombopag and eltrombopag, increase platelet production after binding to the transmembrane region of the TPO receptor and activating signal transduction pathways [103]. Lusutrombopag is currently indicated for chronic liver disease with associated thrombocytopenia [104]. In a study in China, a 60% response rate after treatment with human recombinant TPO (rhTPO) was reported in patients with ITP, who did not develop anti-TPO antibodies, with potential for use in pregnancy [105].

## 6. Conclusions

Attempts to decipher the pathogenesis of ITP have highlighted the fact that there are various pathological pathways that lead to a common feature—thrombocytopenia.

TPO-RAs have been a breakthrough in the management of ITP, and with a deeper understanding of the pathogenesis of ITP, new molecules with surprising mechanisms of action have been developed and will soon be available.

There are still some unresolved issues in ITP, including those related to non-responders, heterogeneity among patients with the same platelet count, patients responding differently to the same treatment, and the exact mechanism that perpetuates disease progression. All of these highlight the crucial need to identify biomarkers that can help clinicians tailor their individualized treatment options.

## Figures and Tables

**Table 1:** Therapeutic targets in ITP management.

Therapy	Mechanism of Action	Drug and Dosage	Durability of Effect	Side Effects and Cautions
Steroids	Broad action on immune cells (macrophages, B and T cells), limiting platelet destruction	Steroid Prednisone/Prednisolone 1–2 mg orally for 1–2 weeks, followed by gradual tapering	Response rate in 60–80% of patients with sustained response after discontinuation in 30–50% of patients	Weight gain, insomnia, acne, mood changes, cushingoid appearance, glucose intolerance, osteoporosis, risk of fracture, gastrointestinal symptoms, neuropsychiatric symptoms
		Dexamethasone 20–40 mg for 4 days every 2–4 weeks; maximum of 4 cycles		
Intravenous Immunoglobulin	Reduction in platelet destruction by inhibiting splenic macrophages Enhancement of antiplatelet antibodies clearance by FcRn saturation	Intravenous Immunoglobulin 1–2 g/kg for 4–5 days	Transient response lasting 1–4 weeks in about 80% of patients	Headache, aseptic meningitis, renal failure
Thrombopoietin receptor agonists	Megakaryocyte-induced increase in platelet production	Eltrombopag 1–10 μg per kilogram, subcutaneously once a week	Response achieved in 1–2 weeks and maintained in 40–60% of patients continuing therapy	Headache, muscle pain, possible increased risk of thrombosis and myelofibrosis
		Romiplostim 25–75 mg orally daily		Gastrointestinal symptoms, hepatocytolysis, cataract, possible increased risk of thrombosis and myelofibrosis—should be taken 4 hr after and 2 hr before food containing cations
		Avatrombopag 5–40 mg orally daily	Response is maintained in 10–30% of patients Response achieved in 1–2 weeks in 65% of patients	Headache, arthralgia, possible increased risk of thrombosis
		Lusutrombopag 3 mg orally daily for 7 days—approved in patients with chronic liver disease who are scheduled for an invasive procedure	78% of patients did not require platelet transfusion prior to the invasive procedure	Increased risk of thrombosis, headache
		Hetrombopag Olamine 2.-7.5 mg orally daily (approved only in China)	Response achieved in 6 days, with a peak at 12–14 days	Hepatocytolysis, hyperuricemia, acute myocardial infarction, anemia
		rhTPO subcutaneously (approved only in China)	Significant increase in platelet count in 7 days	Fever, rash, dizziness, pain at injection site, high blood pressure
Immunosuppressants	Inhibition of T and B cells	Azathioprine 1–2 mg per kilogram orally (maximum 150 mg daily)	Response rate in 30–60% of patients in 1–2 weeks	Weakness, sweating, neutropenia, increased liver function, increased risk of cancer
		Mycophenolate mofetil 500 mg orally twice daily for 2 weeks with gradual increase to a maximum of 1 g twice daily	Response rate in 30–60% of patients in 4–8 weeks	Headache, gastrointestinal symptoms, fungal skin infections, increased risk of cancer
		Cyclophosphamide pulse therapy 1–1.5 g/m^2^ intravenously	Response rate in 85% of patients; mean time to response 7 weeks	Neutropenia, infections, deep venous thrombosis
		Danazol 400–800 orally daily	Response rate in 30–60% of patients within 3–6 months	Hirsutism, acne, amenorrhea, hepatic dysfunction; contraindicated in prostatic cancer
		Dapsone 75–100 mg orally daily	Response rate in 30–60% patients in 3 weeks	Gastrointestinal symptoms, methemoglobinemia, rash, hemolytic anemia (in patients with glucose-6-phosphate dehydrogenase deficiency)
Syk inhibitors	Reduction in platelet destruction by inhibiting macrophage phagocytosis	Fostamatinib 50–150 mg orally twice daily	Response achieved and maintained in 18–43% of patients	Hypertension, nausea, diarrhea, increased liver enzymes
BTK inhibitors	Reduction in platelet destruction by inhibiting macrophage phagocytosis	Rilzabrutinib 400 mg twice daily	Phase 3 Trial (NCT04562766) Response in 40% of patients who received 400 mg twice a day; median time to response 11.5 days	Diarrhea, nausea, fatigue; no grade 3–4 side effects
FcRn blockers	Enhancement of antiplatelet antibody clearance by decreasing peripheral platelet destruction and immune response against megakaryocytes	Rozanolixizumab single-dose 15–21 mg/kg subcutaneous infusion or fractioned doses	Phase 3 Trial (NCT00718692) Better response in single-dose regimen; response in 66.7% patients; median time to response 5–7 days	Headache, diarrhea, pyrexia, nausea, infections
		Nipocalimab Intravenous, subcutaneous	Phase 2 Trial (AIHA, NCT04119050)	
		Efgartigimod 10 mg/kg weekly for 4 weeks, then weekly or every 2 weeks depending on response	Phase 3 Trial (NCT04188379) Response in 51.2% of patients; early platelet count increase	Bruising, headache, hematuria, petechiae
Recombinant immunoglobulin multimers	Reduction in platelet destruction by inhibiting splenic of macrophage phagocytosis Enhancement of antiplatelet antibody clearance by saturation of FcRn	GL-2045 CSL730 M254	Phase 1 Trial	
Complement inhibitors	Decrease complement-dependent cytotoxicity	Sutimlimab (C1s inhibitor) 6.5 g if <75 kg or 7.5 g if ≥75 kg biweekly by intravenous infusion	Phase 2 Trial (NCT04669600) Response obtained in 42% of patients. The median time to first platelet response after the first dose was 22 days	Headache, fatigue
Inhibition of platelet desialylation	Neuramidase-1 inhibitor	Oseltamivir 75 mg twice a day for 5 days	Phase 1/2 Trial (NCT06520049) Higher response rate in patients receiving Oseltamivir and Dexamethasone (86%) than Dexamethasone alone (53%)	
B-cell-depleting therapies—anti-CD20 antibodies	Decrease in short-lived plasma cells by reducing their B-cell precursors, thus decreasing antiplatelet antibodies Restoration of T-cell tolerance	Rituximab 375 mg per square meter of body surface area intravenously weekly for 4 wk (off label use)	Sustained response in 60% of patients at 6 months and 30% at 2 yr; treatment can be repeated	Infusion-related side effects: chills, bronchospasm, neutropenia, hypogammaglobulinemia, serum sickness, increased risk of infections and progressive multifocal leukoencephalopathy; caution in patients with HBV infection or previous HBV infection
		Veltuzumab 80–320 mg 2 doses administered two weeks apart, initially intravenously, then subcutaneously (off label use)	Overall response rate 55%. Median time from first dose to response onset 23 days	Hypersensitivity reaction, fever, body aches, nausea
		Obinutuzumab 1000 mg intravenously at days 1, 8, and 15	Remission rate 100%; only one of four patients relapsed during a follow-up of 15 months	Peripheral neuropathy, fatigue, lymphopenia
Plasma cell-targeting therapies	Decrease in short- and long-lived plasma cells and reducing the antiplatelet antibodies	Proteasome inhibitors: Bortezomib 1.3 mg/m^2^ subcutaneously or intravenously on days 1, 4, and 8 every 3 weeks for a total of 3 cycles	Response rate of 100%; 37.5% complete response (Bortezomib 1.3 mg/m^2^ on days 1, 4, 8, and 11 for 2 cycles)	Peripheral neuropathy, fatigue, lymphopenia
		Anti-CD38 monoclonal antibodies: Daratumumab subcutaneously	Phase II study with safety run-in: 3 patients received 4 weekly subcutaneous daratumumab—2 patients responded, 1 relapsed	Bronchospasm, hypoxia, dyspnea, hypertension, headache
		Mezagitamab Subcutaneously	Phase 2 trial (NCT042789) no results posted	Headache, dizziness, chills, mild injection site reactions
B-cell- and plasma cell-targeting therapies	Decrease in short- and long-lived plasma cells, thus decreasing antiplatelet antibodies	Anti-CD19 - Inebilizumab - Obexelimab	No clinical trials for ITP	
T-cell-targeting therapies	Inhibition of effector T cells Inhibition of follicular helper T cells	Anti-CD154 Rupulizumab (hu5c8) 20 mg/kg once every 4 weeks for 12 weeks	Response in 43% of patients	Thromboembolic events
		Toralizumab (IDEC-131) 5–20 mg/kg	Response in 16% of patients	Thromboembolic events
		Letolizumab (BMS-986004) 75–1500 mg intravenously	Response in 20–40% of patients	Anemia, hemorrhage, fatigue, headache, body ache
		Anti-CD40 BI 655064	No available results from clinical trials for ITP	Infection, opportunistic infections, neutropenia, lymphopenia, alopecia
	Restoration of regulatory	IL-21 inhibitors	No clinical trials for ITP	
		Low-dose IL-2 1 million IU/day 5 days per week for 2–4 weeks	Phase 2 Trial (NCT01988506) Improvement in platelet count in 2 of 3 patients	Injection site reaction0061
Epigenetic modulation		Chidamide (Tucidinostat) 2.5 or 5 mg orally, twice a week for four weeks, one cycle	Phase 2 Trial (NCT03838354)	Diarrhea, fatigue, nausea
		Low-dose decitabine 3.5 mg/m2 IV for 3 consecutive days, repeated every 28 days for three cycles	Complete response in 17.78%; partial response in 33.33%. Median time to response 28 days	Nausea, fever; no grade 3–4 adverse events
Splenectomy	Reduction in platelet destruction by inhibiting splenic macrophage phagocytosis Removal of the maintenance site of the autoimmune response		Favorable response obtained in 89% of patients	Postoperative complications, major bleeding higher risk for the elderly group, infections, thrombotic events

Abbreviations: FcRn = neonatal Fc receptor; rhTPO = recombinant human thrombopoietin; Syk = spleen tyrosine kinase; BTK = Bruton kinase; HBV = hepatitis B virus; IL = interleukin; ITP = immune thrombocytopenia; CD = cluster of differentiation.
